# Molecular modeling and *in silico *characterization of *Mycobacterium tuberculosis *TlyA: Possible misannotation of this tubercle bacilli-hemolysin

**DOI:** 10.1186/1472-6807-11-16

**Published:** 2011-03-28

**Authors:** Nelson E Arenas, Luz M Salazar, Carlos Y Soto, Carolina Vizcaíno, Manuel E Patarroyo, Manuel A Patarroyo, Arley Gómez

**Affiliations:** 1 Departamento de Química, Facultad de Ciencias, Universidad Nacional de Colombia, Carrera 45 No. 26-85 Bogotá, DC. Colombia; 2 Fundación Instituto de Inmunología de Colombia (FIDIC), Carrera 50 No. 26-20 Bogotá, DC. Colombia; 3 Universidad del Rosario, Carrera 24 No. 63C-69 Bogotá, DC. Colombia; 4 Centro de Investigaciones Biomédicas, Universidad del Quindío, Armenia, Colombia. Carrera 15 calle 12 Norte. Armenia. Quindío. Colombia

## Abstract

**Background:**

The TlyA protein has a controversial function as a virulence factor in *Mycobacterium tuberculosis *(*M. tuberculosis*). At present, its dual activity as hemolysin and RNA methyltransferase in *M. tuberculosis *has been indirectly proposed based on *in vitro *results. There is no evidence however for TlyA relevance in the survival of tubercle bacilli inside host cells or whether both activities are functionally linked. A thorough analysis of structure prediction for this mycobacterial protein in this study shows the need for reevaluating TlyA's function in virulence.

**Results:**

Bioinformatics analysis of TlyA identified a ribosomal protein binding domain (S4 domain), located between residues 5 and 68 as well as an FtsJ-like methyltranferase domain encompassing residues 62 and 247, all of which have been previously described in translation machinery-associated proteins. Subcellular localization prediction showed that TlyA lacks a signal peptide and its hydrophobicity profile showed no evidence of transmembrane helices. These findings suggested that it may not be attached to the membrane, which is consistent with a cytoplasmic localization. Three-dimensional modeling of TlyA showed a consensus structure, having a common core formed by a six-stranded β-sheet between two α-helix layers, which is consistent with an RNA methyltransferase structure. Phylogenetic analyses showed high conservation of the *tlyA *gene among *Mycobacterium *species. Additionally, the nucleotide substitution rates suggested purifying selection during *tlyA *gene evolution and the absence of a common ancestor between TlyA proteins and bacterial pore-forming proteins.

**Conclusion:**

Altogether, our manual *in silico *curation suggested that TlyA is involved in ribosomal biogenesis and that there is a functional annotation error regarding this protein family in several microbial and plant genomes, including the *M. tuberculosis *genome.

## Background

Tuberculosis (TB) is an infectious disease that mainly afflicts populations in third-world countries. Although most infected people will never develop an active form of the disease, the global TB death rate is substantial, being around two million people per year. The situation has been further exacerbated by the emergence and spread of extremely resistant *Mycobacterium tuberculosis *strains (XDR) [1] which, together with the lack of effective antibiotics, urges the development of new alternatives for controlling this worldwide public health threat. Consequently, most research efforts have focused on understanding *M. tuberculosis *biology with the aim of identifying new therapeutic and vaccine targets.

Current management of TB cases consists of a two-month intensive treatment comprising first-line drugs such as Rifampicin (RIF), Isoniazid (INH), Pyrazinamide (PZA) and Ethambutol (EMB)/Streptomycin (SM) which seeks to ensure that mono-resistant strains do not proliferate [2]. When RIF and INH are ineffective, standard treatment guidelines recommend a combination of second-line drugs [3]. Among these therapeutic choices, aminoglycosides such as Capreomycin (CMN) and Viomycin (VMN) are given in combination with other antibiotics to treat multidrug-resistant strains (MDR); they are also effective against non-replicating *M. tuberculosis *strains, as well as being useful in the treatment of latent TB infections [4]. CMN- and VMN-resistant bacilli are classified as XDR-TB strains and are a cause of major concern since these drugs have more toxic side effects and might result in higher death rates, especially among HIV-infected persons [1].

Controversially, *M. tuberculosis tlyA *gene product has been annotated both as a virulence factor, due to its ability to lyse red blood cells, as well as a protein involved in ribosome biogenesis [5,6].

TlyA has been assumed to induce hemolytic activity due to its high amino acid similarity (37.3%) to the pore-forming hemolysin/cytotoxin virulence determinant from *Serpulina hyodysenteriae*. Notably such activity was only experimentally demonstrated with bacterial lysates [6] and another study has even suggested that TlyA could be associated with hemolysin expression in *Escherichia coli *[7,8]. Additionally, recent study showed that a concentration-dependent hemolysis of rabbit and human erythrocytes to be induced on incubation with the recombinant Rv1694 protein (the putative *M. tuberculosis *TlyA) when over-expressed in *E. coli *[8]. The article reported hemolysis inhibition using specific antiserum against the same protein as well as the presence of the recombinant protein on *E. coli *cell wall; these data were supportive of TlyA's hemolytic property, combined with a preliminary *in silico *analysis. Rv1694 oligomers were also observed on lysed erythrocyte membranes, as well as the susceptibility of Rv1694-expressing *E. coli *to CMN [8]. Despite such thorough *in vitro *analysis in *E. coli*, the functional activities suggested for Rv1694 have still not been clearly demonstrated when using *M. tuberculosis *mutant strains.

By contrast with the proposed hemolytic function, Johansen *et al.*, [5] have reported that *M. tuberculosis *H37Rv and Beijing TlyA confer susceptibility to CMN and VMN. These antibiotics are structurally similar and share the same mechanism of action, both inhibiting bacterial proliferation by blocking the peptidyl transferase reaction. VMN hinders A-site and P-site tRNA positioning on the 50S ribosomal subunit, therefore hampering mRNA translation [9]. CMN resistance arises due to mutations in the *tlyA *gene which encodes a RNA 2'-O-methyltransferase (2'-O-MTase) that methylates ribose moieties from nucleotide C1409 in 16S rRNA and C1920 in 23S rRNA. The absence of these rRNA methylations confers resistance against CMN and VMN given that binding sites for these antibiotics are delineated by the localization of such methylated riboses [5]. Additional evidence has been provided by mutant complementation, showing that CMN susceptibility is restored by introducing an active copy of the *tlyA *gene [10]. TlyA methylation clearly enhances CMN and VMN susceptibility and is responsible, at least in part, for their efficacy against mycobacterial infections as drug binding is impaired when the enzyme is not produced. Resistance thus emerges when TlyA activity is lost. These studies show the need for a reevaluation of TlyA's function in virulence [5,11].

## Results

### TlyA related sequences

**Figure 1:**
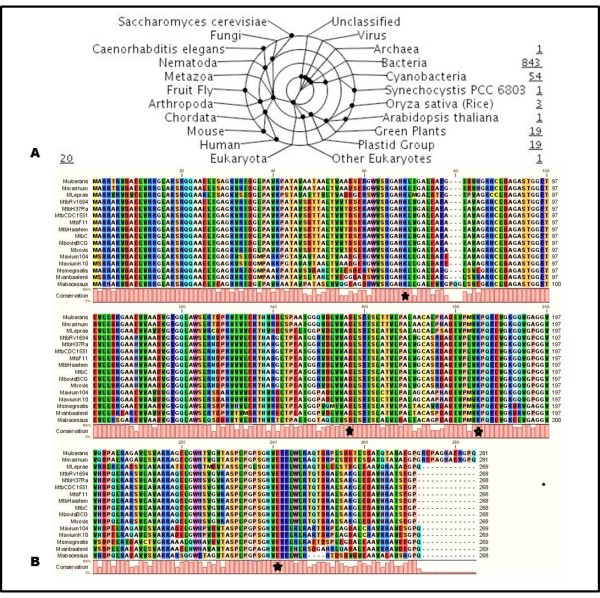
**Hemolysin A family (InterPro: IPR004538) taxonomic coverage of 864 proteins**. (A). Taxonomy-tree nodes are placed on the inner circles and radial lines lead to the description for each node. Proteins annotated by taxonomic division are numbered [http://www.ebi.ac.uk/interpro/IEntry?ac=IPR004538]. (B). Multiple sequence alignment of TlyA protein homologs in *Mycobacterium*. Sequences are colored according to amino acid physicochemical properties; the catalytic tetrad is marked by black asterisks (K69-D154-K182-E238). Similarity values for each amino acid are represented by bars (pink). Organism names correspond to nomenclature found in the tuberculosis database [http://www.tbdb.org/] (CLC sequence viewer alignment representation).

The BLAST search for TlyA-related sequences in the TB and Genbank databases showed that although TlyA-related sequences are found mainly in prokaryotic organisms, homologous protein sequences are also present in eukaryotic organisms such as plants and algae (Figure 1A).

These *M. tuberculosis*-related TlyA protein sequences are mainly classified as hemolysin A inside the FtsJ-like class based on domain similarity and have been annotated as such in Genbank, EMBL, UniProt and SWISS-PROT databases according to sequence similarity with *Serpulina hyodysenteriae *TlyA annotated as being a hemolysin. Paradoxically, although hemolysin A contains similar domain architecture to TlyA, it is predicted to be a rRNA methylase instead of a contact-dependent hemolysin (Table S1, Additional file 1).

TlyA amino acid sequences from *M. tuberculosis *H37Rv, H37Ra, CDC1551, *M. bovis *and *M. bovis*-BCG reference strain alignment showed a 100% identity with the Harleem, F11 and C *M. tuberculosis *strains (Figure 1B). The same conservation pattern was obtained when the corresponding nucleotide sequences were aligned (> 99% identity).

Similarity percentages found in the multiple amino acid alignment of *Mycobacterium *species different to *M. tuberculosis*, such as *M. smegmatis *(76%), *M. abscessus *(72%), *M. leprae *(78%), *M. avium *(79-80%) and *M. vanbaalenii *PYR-1 (76%), showed high TlyA conservation at genus level. A glutamine residue insertion was found at position 269 in the protein's C-terminal region in all the aforementioned species, while a 12-residue insertion was found only in *M. ulcerans *(81%) and *M. marinum *(81%), which are pathogenic mycobacteria (Figure 1B).

### TlyA transcriptional unit revealed some rearrangements in the *Mycobacterium IprJ-recN *operon

**Figure 2:**
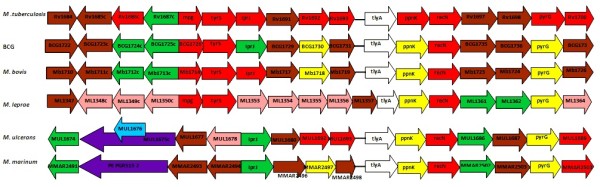
***tlyA *transcriptional unit gene syntheny**. Genes are represented as arrows and are drawn according to transcriptional orientation and genome functional annotation. Arrow color representation for gene functional annotation is as follows: green for cell wall synthesis, red for signal transduction, brown for evolutionarily conserved, yellow for intermediary metabolism, white for *tlyA*, pink for pseudogenes, purple for PE/PPE and blue for IS/phages.

From the synteny of the *IprJ-recN *operon from *M. tuberculosis*, *M. bovis *BCG, *M. bovis*, *M. leprae*, *M. ulcerans *and *M. marinum *(Figure 2) it was evident that almost all gene products, except for *tlyA*, are involved in intermediary metabolism, cell wall biosynthesis and signal transduction rather than virulence. Moreover, no trafficking proteins were found within the operon or near *tlyA *that could be suggestive of TlyA transportation either to the cell membrane or the extracellular milieu.

The *IprJ-recN *operon also had common gene organization in *M. tuberculosis *and *M. bovis*, while in *M. leprae *most genes have lost their function (i.e. have become pseudogenes). Such high rate of loss of function in genes involved in intermediary metabolism, cellular respiration and cell-wall biosynthesis may suggest that the loss of such genes confers an adaptive advantage on *Mycobacterium *during host cell infection or transmission [12]. No other genes reported to be involved in bacilli virulence were found within this operon.

### TlyA three-dimensional structure

The three-dimensional structure of a putative hemolysin from *Streptococcus thermophilus *(PDB ID: 3HP7 chain A, at 1.53 Å resolution) was used as template for homology modeling. The obtained identity (37.2%, RMSD 0.44 Å, E-value 4.7e-26) suggested that the selected hemolysin structure was a suitable template for TlyA, and that the obtained modeled structure could reflect an experimentally-obtained *M. tuberculosis *TlyA structure.

Although this protein seemed to be a hemolysin, its domain architecture was also similar to RNA methyltransferase family, fibrillarin homologues and TlyA proteins. A consensus sequence was established which showed the presence of a common core, comprising six β-sheets, the first five of which were found parallel and the sixth anti-parallel between two layers of α-helices and random coiled regions. Such structural organization is commonly referred as to an AdoMet-dependent methyltransferase (MTase) fold [13,14].

**Figure 3:**
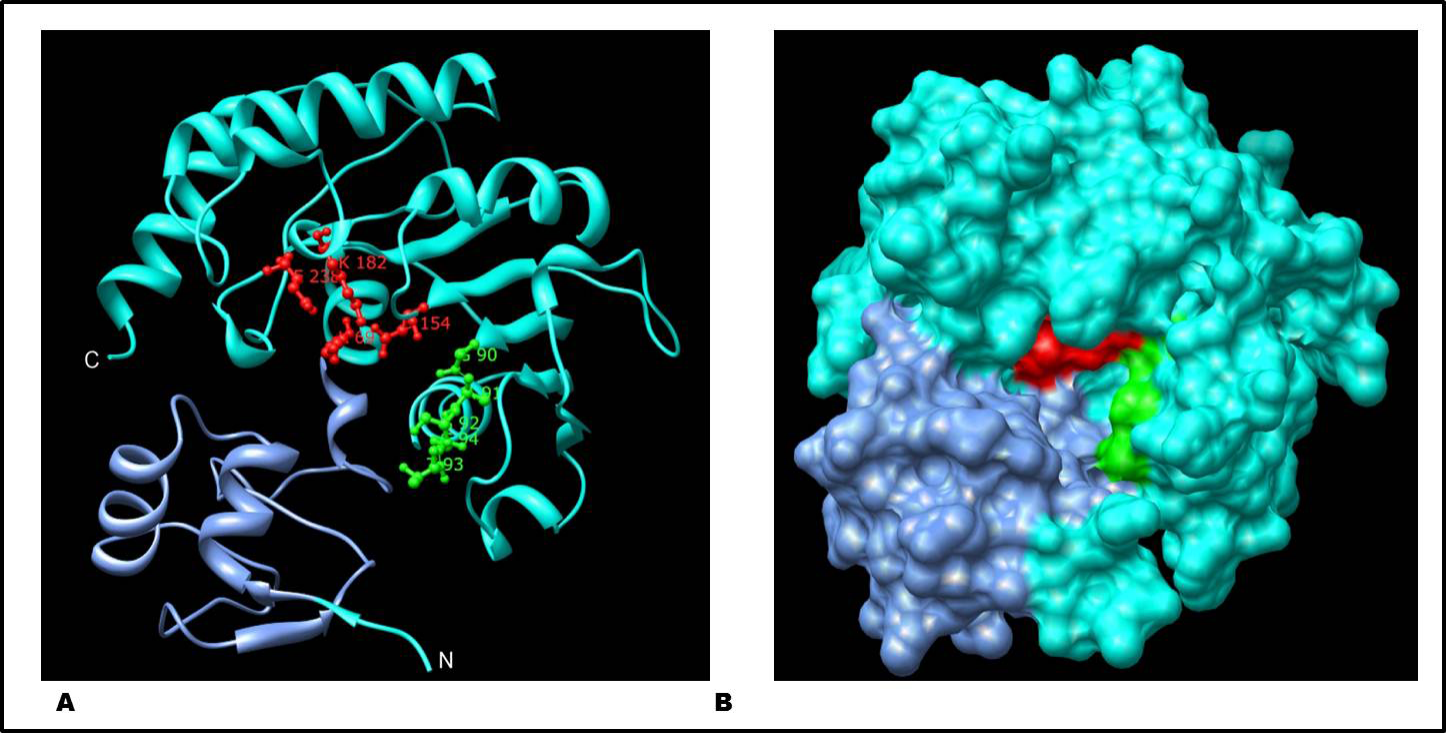
**Modeled spatial configuration built using UCSF Chimera**. Ribbon (A) and space-filling (B) representations of TlyA catalytic tetrad (red), AdoMet binding motif GASTG90-94 (green) and S4 RNA binding domain (blue). Structural model was stored on Protein Model Database [PMDB: PM0076044, http://mi.caspur.it/PMDB/].

TlyA has an overall globular fold and comprises two domains exhibiting different spatial arrangements. The smaller domain, named S4, is N-terminally located and is overlapped by the catalytic domain which exhibits structural similarity to various unrelated RNA-binding proteins, while the large catalytic domain, named FtsJ-like methyltransferase domain is common to 2'-O-MTases and exhibits a α/β fold with a deep pocket (Figure 3).

The S4 domain consists of 64 residues, starting at A-5 and ending at H-68. This domain is structurally formed by one β-sheet and three α-helices predominantly composed of positively charged residues (11 out of 47 residues) whose probable function is to mediate RNA binding and provide stability. In line with this, the RNABindR server identified three short sequences (1-MARRAR-6, 13-RRGLARSRQQ-22 and 31-KVR-33) inside the S4 domain that might be implicated in RNA binding (Figure 3). The S4 domain has been specifically identified in some bacterial and eukaryotic ribosomal proteins, pseudouridine synthases, RNA methylases, bacterial tyrosyl-tRNA synthetases and may also be involved in translation regulation [15].

**Figure 4:**
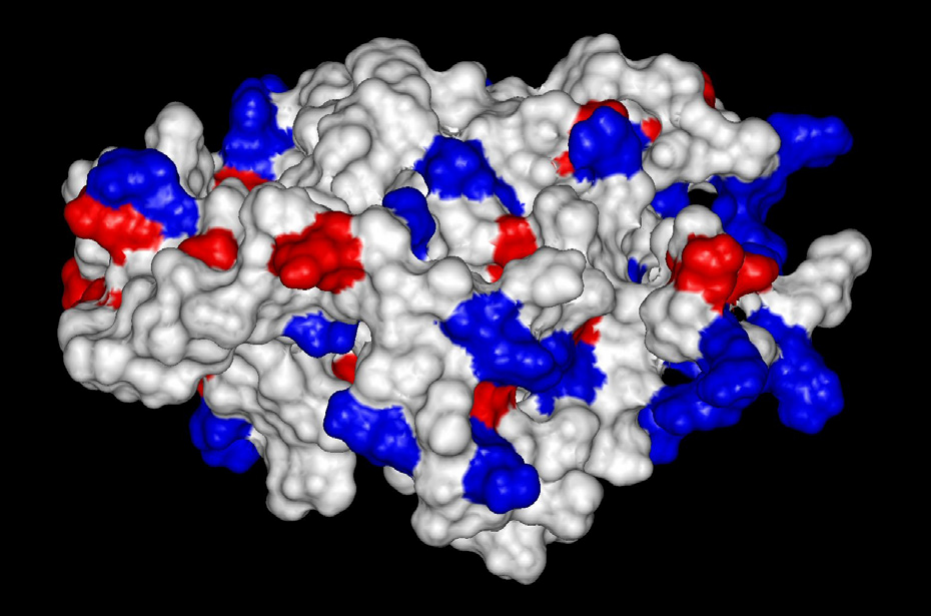
**Diagram of charge distribution on TlyA Connolly surface**. Three-dimensional modeled structure according to positive (blue) and negative (red) charge distribution on the protein surface.

The analysis of the protein's electrostatic surface showed that positively charged residues were localized towards the N-terminal extreme, forming bulges and long finger-like projections that probably extend into the rRNA core to stabilize its structure (Figure 4). In fact, charged residues like R and K exhibited the highest interface propensities, which is consistent with their ability to participate in interactions both with nucleotide bases and the negatively charged RNA phosphate backbone [16].

The FtsJ-like methyltransferase domain is located between residues 62 and 247 and includes the catalytic tetrad responsible for TlyA's enzymatic activity [14]. Moreover, the FtsJ-like methyltransferase domain exhibits a Rossmann fold composed of five parallel β-strand layers linked by two α-helices following an α/β secondary structure topological order [17].

Our comparative analysis of TlyA with the Fibrillarin family showed the presence of a GASTG AdoMet binding motif comprising residues 90-94 and a D located at position 112 juxtaposed to the above mentioned motif (Figure 3A), which might stabilize TlyA coenzyme binding [13]. AdoMet was located between β-strands 4 and 5 within the inner depression inside the catalytic pocket formed by GASTG residues' main-chain NH groups (Figure 3B). Moreover, substrate positioning might be driven by the Rossmann configuration fold and the pertinent AdoMet-binding/catalytic sites, suggesting substrate selectivity in the small ribosomal subunit [17]. Likewise, domain structure was in agreement with S-adenosyl-methionine (AdoMet)-dependent methyltransferases. AdoMet binding and affinity might indicate how an enzyme such as TlyA may methylate the two adjacent ribose rings on both ribosomal subunits. In this reaction S-adenosyl-methionine (SAM) acts as methyl donor, as it is converted into S-adenosyl-l-homocysteine (SAH); TlyA could thus be also classified as being an RNA-AdoMet-MTase class I [18].

This hypothesis is supported by the high structural similarity (98.1%) between TlyA and FtsJ RNA-MTase complexed with AdoMet (ligand-binding template PDB entry 1EJ0) (RMSD 2.98, Similarity score: 368.77 E-value: 1.30E-06) (Figure S1, Additional file 2). Two expected structural motifs were also identified in this structure which was formed by six buried residues between G-90 and F-96 which are pivotal for AdoMet interaction and therefore conserved in *Mycobacterium *TlyA proteins (Table 1).

**Table 1:** Nest analysis

Score	Residue range	Residue	Ramachandran region	Solvent accessibility	Cleft	Depth in cleft	Residue conservation
5.50	Thr93-Phe96	Thr93	RIGHT	0.00%	1	14.74	1.00
		Gly94	LEFT	0.22%	1	15.55	1.00
		Gly95	RIGHT	2.72%	1	16.88	1.00
		Phe96	RIGHT	1.03%	1	19.99	1.00
4.67	Gly90-Ser92	Gly90	LEFT	4.04%	1	14.84	1.00
		Ala91	RIGHT	0.00%	-	-	1.00
		Ser92	-	0.00%	1	18.32	1.00

Nest analysis of two structural motifs detected in TlyA functionally important regions for AdoMet binding region. Results were obtained on Profunc server [43].

The TlyA model was validated by using the RAMPAGE server which evaluates a protein's 3D-structure based on the stereochemistry quality of torsion angles and geometry [19]. The Ramachandran plot showed 95.1% feasibility (253 aa) in favorable positions and 3.8% (10 residues) in acceptable regions, with just 1.1% in an outlier region (3 residues), supporting the high quality of the 3D-model (Figure S2, Additional file 3).

This *in silico *structure analysis of *M. tuberculosis *proteins allowed a deeper understanding of TlyA's function and challenges the initially described function of TlyA-related proteins. Rather than its hypothetical function as a hemolysin, our data suggested functions as an MTase based on *in silico *prediction.

### Predicting subcellular localization

Subcellular localization was predicted with TBpred [http://www.imtech.res.in/raghava/tbpred/] and the results suggested TlyA localization in the cytoplasmatic compartment (Table 2), also supporting the fact that TlyA acts as a methyltransferase [5]. A recent mycobacterial protein analysis led to ascertaining several predictors' reliability for subcellular localization, providing highly accurate results for Gpos-PLoc and PA-SUB v.2.5 and SignalP 2.0 as a confirmatory tool [20]. However, in this study, PA-SUB v.2.5 predicted TlyA as being an extracellular protein (72.84%), which could have been biased as the training strategy used involved results from the SWISS-PROT [21] database where TlyA is mis-annotated. Likewise, in agreement with BLAST scores for the query sequence (Rv1694), 26 out of 28 homologous proteins were designated as putative ribosomal RNA methyltransferases and RNA binding proteins. Only one submitted sequence seemed to be similar to hemolysin A (the *S. hyodysenteriae *sequence).

**Table 2:** TlyA subcellular localization prediction through 9 available servers for prokaryotic proteins

Tool	Subcellular localization	Value	Reliability Index	Method	Overall prediction accuracy	**Ref**.
TBpred	Cytoplasmic	1.365	Higher value above 0 or 1	SVM/PSSM, MEME/MAST, HM.	82.51, 80.39 and 86.62%	24
Gpos-PLoc	Cytoplasmic	ND	ND	By fusing PseAAC	> 80%	25
PSORTb	Cytoplasmic	8.870	≥ 7.5	SCL-BLAST, SVM, Motif and Profile Analysis	96%	26
CELLO	Cytoplasmic	3.809	Higher value	SVM based on multiple n-peptide composition	89%	27
LOCtree	Cytoplasmic	8.000	>3	SVM	ND	28
SubLoc v1.0	Cytoplasmic	5.000	5	SVM	96%	29
PA-SUB	Extracellular	72.84%	Higher percent	SVM	> 92%	30
MemType-2L	Non membrane protein	ND	ND	Pse-PSSM	92.7	31
TMBETA-SVM	Non membrane protein	0.636	ND	SVM	92%	32

ND: non defined value. Pse-PSSM: Pseudo Position-Specific Score Matrix. SVM: support vector machines. PSSM: Position-Specific Scoring Matrix. MEME/MAST: Multiple Em for Motif Elicitation/Motif Alignment and Search Tool. HM: Hybrid module. PseAAC: Pseudo Amino Acid Composition.

### Evolutionary insights into *tlyA *genes

**Figure 5:**
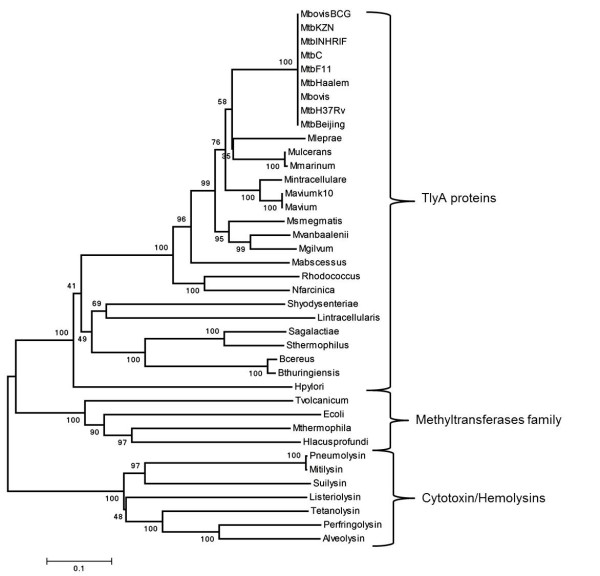
**Evolutionary relationships of 34 taxon at amino acid level**. Evolutionary relationships were inferred by the NJ method. The optimal tree had 5,628 branch length sum 5,000 replicates were carried out; bootstrap values are shown at each cluster.

Evolutionary analysis of *tlyA *genes from 16 mycobacterial species, 8 species taxonomically related to *Mycobacterium*, 4 Adomet-RNA MTases and 7 PFP showed similar topology by both Neighbor-joining (NJ) and Maximum Parsimony (MP) methods, suggesting that the phylogenetic relationship observed was highly reliable (Figure 5).

The consensus tree showed the *tlyA *gene in *M. tuberculosis *H37Rv, CDC1551, C, Haarlem, Beijing, KZN and INH-RIF strains as well as in the *M. bovis *AF2122 and BCG strains to fit in a monophyletic cluster. The *tlyA *tree exhibited short genetic distances (less than 1%) for *M. tuberculosis *complex species, including those separated according to clinical considerations.

Phylogenetic analysis showed that *Mycobacterium tlyA *genes are closely related to those of *Nocardia *and *Rhodococcus *genus; such branching patterns were supported by high bootstrap values and agreed with the results obtained from 16S rDNA phylogeny. No differences were observed between antibiotic susceptible and resistant *M. tuberculosis *strains. *Mycobacterium *TlyA proteins fell into 4 groups; each group formed a well-delineated branch supported by high bootstrap values. *M. abscessus *was the most distant and TlyA-ancestral species from the *Mycobacterium *genus compared to the closest species to *M. tuberculosis *(*M. ulcerans, M. marinum *and *M. leprae*). *Serpulina hyodysenteriae *and *Lawsonia intracellularis *LsaA antigen sequences were included due to their sequence identity to *M. tuberculosis *TlyA (26.5%), sharing a common ancestor, although having experimentally-attributed different roles [6,22-24] (Figure 5).

Pattern branching of the phylogenetic tree showed that the TlyA protein formed a monophyletic cluster with AdoMet RNA MTases, suggesting a common evolutionary origin; these findings might also imply a similar functional role in translational machinery. PFP formed a distant cluster where diverse proteins were clustered on a branch with the most divergent proteins (Figure 5).

The synonymous and non-synonymous substitutions were calculated to identify the action of neutral, positive or purifying selection on *tlyA *genes; their synonymous, Pi(a), and non-synonymous diversity, Pi(s), was 0.672 and 0.132, respectively, corresponding to a 0.196 Pi(a)/Pi(s) rate which can be inferred as purifying selection acting on this gene among *Mycobacterium *species. This suggests that conservation at sequence level might show *Mycobacterium tlyA *gene homogeneity and show low inter-specific variation between the species compared in this study.

## Discussion

The aim of the present study was to describe structural and phylogenetic features of *M. tuberculosis *TlyA, using bioinformatics tools. The structural and phylogenetic analysis shown here highlights the need for re-annotating this protein family and redefining it as an RNA-binding FtsJ-like methyltransferase in bioinformatics databases; this is based on the high degree of amino acid sequence similarity found in the *Mycobacterium *species analyzed here. It can be surmised that physical organization, domain spatial arrangement and protein folding on TlyA is structurally conserved.

Previous sequence analysis of 2'-O-MTase families has indicated that they use a similar catalytic mechanism and have inherited a common function from the same ancestral ribose 2'-O-MTase. Feder *et al.*, suggested that TlyA is a 2'-O-MTase, identifying a catalytic tetrad consisting of lysine-69, aspartic acid-154, lysine-182 and glutamic acid in position 238 (K69-D154-K182-E238) [14]. Based on amino acid sequence alignment, our comparative analysis with the RrmJ family and other related RNA 2'-O-MTases [13,14,18] showed changes in D154-K182-E238 residue positions (Figure 1). However, despite being located in different sequence positions, the scaffold formed by these residues was maintained at the catalytic site in the modeled protein 3D structure without affecting protein function [25].

Despite the diversity of existing MTases, the majority maintain the same catalytic mechanism where K-182 binds to a hydrogen from the ribose 2-OH' group; this nucleophilic attack generates a transition state (S_N_2-like state). The lysine side chain shifts E-238 so that its negatively charged carboxyl group becomes available to promote the attack of the methyl donor [18]; furthermore, this conserved amino acid residue pattern was also observed in our evolutionary trace analysis.

### TlyA three-dimensional structure resembled an RNA methyltransferase

The *S. hyodysenteriae *TlyA postulated mechanism for hemolysin action is said to be similar to that for pore-forming protein (PFP) mechanisms [6]; TlyA folding, however, is inconsistent with PFPs due its lack of membrane-binding domains, cholesterol-recognition and insertion motifs, and other characteristic features such as a conserved undecapeptide (ECTGLAWEWWR) near the C- or N- terminal ends. It also lacks repeat domains in the toxin (RTX) and calcium-binding glycine-rich motifs necessary for oligomerization [26]. This *in silico *structure analysis of *M. tuberculosis *proteins led to a deeper understanding of TlyA's role and challenged the initially described function for TlyA-related proteins.

A combination of bioinformatics analysis and experimental structure elucidation may suggest alternative functional roles for previously-annotated enzymes, as with (SAM)-dependent methyltransferase, phosphatase and N-acetyltransferase structures from *M. tuberculosis *[11,27,28]. Theoretical evidence has thus been provided for TlyA's role in RNA modification instead of it just being a hemolytic factor.

### Subcellular localization suggested a cytoplasmatic localization consistent with ribosome-associated activity

Predicting subcellular localization is important since several characteristics can be deduced, such as protein function and genome annotation. It also aids experimental design for proteomics platforms, particularly for identifying new candidates for vaccine development and drug targets [29,30]. Protein sequence analysis did not reveal a signal peptide, suggesting that TlyA is not secreted; however, some authors have described TlyA as being a probable PFP [6], supported by the presence of over-expressed TlyA on the *E. coli *wall surface [8].

TlyA lacks transmembrane helices and β-barrels (2.877 value), suggesting that this protein might not be a membrane-embedded or contact-dependent hemolysin, as has previously been proposed [6,23]. TlyA also has a hydrophobic region in the C-terminal domain (residues 115-133) which may not form a membrane-spanning α-helix. Nonetheless, such a region might allow the adenine present in AdoMet to fit properly inside the catalytic pocket, thus suggesting a different role for these hydrophobic residues. Virulence-related functional annotation, based on all the above-mentioned data, remains controversial.

Conservation of *tlyA *genes and functions was expected according to our phylogenetic and evolutionary trace analyses, the initial hypothesis being that homologous proteins have related functions. However, the extent to which this is true has not been assessed in detail up to now; annotation based on protein homology might thus lead to finding unexpected variations in function.

## Conclusions

The main concern of our work was to decipher structural information about the TlyA family by homology comparison. Our structural analysis and finding residue conservation within the active site supports recent experimental work [8] about TlyA's role as RNA 2`-O-MTase in *M. tuberculosis*. This is further supported by the domain composition also suggesting methyltransferase activity and RNA-binding. The distribution of charged residues also defines a likely RNA interaction instead of hemolytic activity, little similarity being exhibited with hemolysin proteins, perhaps representing inaccurate annotations in several databases, as observed with other protein families [31].

Since genome annotation is based on sequence comparison, it is the most commonly used approach for determining functional homology. Annotations are assigned if proteins surpass similarity cut-off and the propagation of original errors could be increased by each new entry. In such cases, protein function needs to be inferred from their common three-dimensional structures and manual curation.

## Methods

### Search for TlyA related sequences

Sequences homologous to *M. tuberculosis *TlyA (Rv1694; GenBank: CAA66941) in other *Mycobacterium *spp. were found by screening the TB-Database [http://www.tbdb.org/]. Protein and gene sequence similarity was analyzed by BLAST tools [32] for searching the NCBI non-redundant sequence database [http://www.ncbi.nlm.nih.gov/] and a homology comparison was made using BLASTp for the PDB database. The TlyA-like sequences so obtained were aligned using the CLUSTALW Multiple Sequence Alignment program [http://align.genome.jp/] [33] and manually edited in BIOEDIT [34]. The alignment was visualized using the CLC sequence alignment viewer v6.0 [http://www.clcbio.com] [32] to obtain similarity scores for each amino acid position. The *M. tuberculosis IprJ-recN *operon genes (*IprJ*, *Rv1691*, *Rv1692*, *Rv1693*, *tlyA*, *ppnK *and *recN*), where *tlyA *gene is located, were all searched within the *M bovis*, *M bovis *BCG, *M. leprae*, *M. ulcerans *and *M. marinum *genome databases for visual examination of the transcriptional unit.

### Fold-recognition and domain analysis

This protein's domain composition was analyzed using the SMART-Simple Modular Architecture Research Tool [http://smart.embl-heidelberg.de] [35] in combination with the Pfam database [http://www.sanger.ac.uk/Software/Pfam/] [36].

An *M. tuberculosis *TlyA (Rv1694) secondary structure consensus was built based on the predictions obtained with SAM [37], PSIPRED [38] and JNet secondary structure prediction [39] servers. This consensus allowed poorly and highly structured regions to be compared for selecting the best tridimensional (3D) structure model according to structural homology and folding prediction.

### Protein 3D-structure prediction

An *M. tuberculosis *TlyA structural model was obtained from its amino acid sequence by using SWISS MODEL [http://swissmodel.expasy.org/][40] and Protein Homology/analogY Recognition Engine (PHYRE) [http://www.sbg.bio.ic.ac.uk/phyre/] prediction servers [32,41]; the obtained models were classified according to identity percentages. Both, protein structure and function models (3HP7, 1QD7, 1Q8K, 3DOU, 2PLW, 1FJG, 1EJ0 PDB entries) were compared to the secondary consensus sequence obtained as described above. Structure refinement and minimization were carried out using UCSF CHIMERA program [42], and the resulting 3D-model was submitted to ProFunc server [http://www.ebi.ac.uk/thornton-srv/databases/profunc/][19,32,43] to predict biochemical functions and structural motifs. The obtained 3D-model was stereo-chemically evaluated on RAMPAGE server [19] which provides a score based on proline and glycine preferential positions according to a Ramachandran plot.

### Subcellular localization prediction

The Phobius server [44] was used to predict the presence of the signal peptide and transmembrane α-helices within the *M. tuberculosis *TlyA amino acid sequence. TlyA's subcellular localization was predicted using the TBpred server [29], a specific tool for mycobacterial proteins. Results were compared to subcellular localization predictions obtained from Gpos-PLoc [30], PSORTb v2.0.4 [45], PA-SUB [46], CELLO v2.5 [47], LOCtree [48], SubLoc v1.0 [21], MemType-2L [49] and TMBETA-SVM [50]. The position of RNA binding residues was predicted using the RNAbind server [51].

β-barrel structures were predicted with PRED-TMBB [http://biophysics.biol.uoa.gr/PRED-TMBB/input.jsp] using a 2.965 threshold value [52].

### Phylogenetic and evolutionary trace analysis

The molecular phylogenetic tree for TlyA was built with Neighbor Joining (NJ), using p-distance as substitution model, and Maximum Parsimony (MP) methods in MEGA Version 4.0, with 5,000 iterations for calculating bootstrap confidence levels [53]. Phylogenetic tree construction included the sequences for *Mycobacterium *TlyA proteins and/or putative cytotoxin/hemolysins reported in the TB-database for the following 11 mycobacterial species, including 14 different strains. Pertinent sequences were found in 21 *M. tuberculosis*-related species (Table S1, Additional file 1).

Evolutionary Trace Report Maker [54] and Evolutionary Trace Analysis [55] and DnaSP [56] were used for identifying residues under evolutionary pressure and substitution rates were determined using MEGA 4.0.

## Authors' contributions

NEA, CV and LMS wrote the manuscript, validated the tools and carried out the data analysis and interpretation. CYS, MEP, MAP and AG contributed to the methodological design, supervised its development and critically revised the manuscript's content. All authors read and approved the final version of the manuscript.

## Supplementary Material

Additional file 1 **Table S1**. Descriptions of TlyA proteins used in the phylogenetic analysis. Protein sequences were gathered from the GenBank [http://www.ncbi.nlm.nih.gov/] by BLAST search [32]. Click here for file

Additional file 2 **Figure S1**. Sequence alignment and secondary structure representation for TlyA and Ftsj-like RNA methyltransferase complexed with AdoMet, defined as the ligand-binding template [PDB:1EJ0] [31] showing the matched residues with the template. Alpha helices are represented by red spirals, beta strands by yellow arrows and random structures by straight lines. The red bar indicates the region with greater structural similarity (152 residues-long). Amino acid regions sharing the highest similarity are enclosed in boxes. Click here for file

Additional file 3 **Figure S2**. Ramachandran plot of TlyA three-dimensional modeled structure. Dispersion zones are shown in blue and orange representing proline and glycine favored and allowed regions (Figure obtained by RAMPAGE server). Click here for file
