# Supplementary figures and images for: Molecular modeling and in silico characterization of Mycobacterium tuberculosis TlyA: Possible misannotation of this tubercle bacilli-hemolysin
Source: BMC Struct Biol. 2011 Mar 28;11:16. doi: 10.1186/1472-6807-11-16 (PMC3072309; doi:10.1186/1472-6807-11-16)

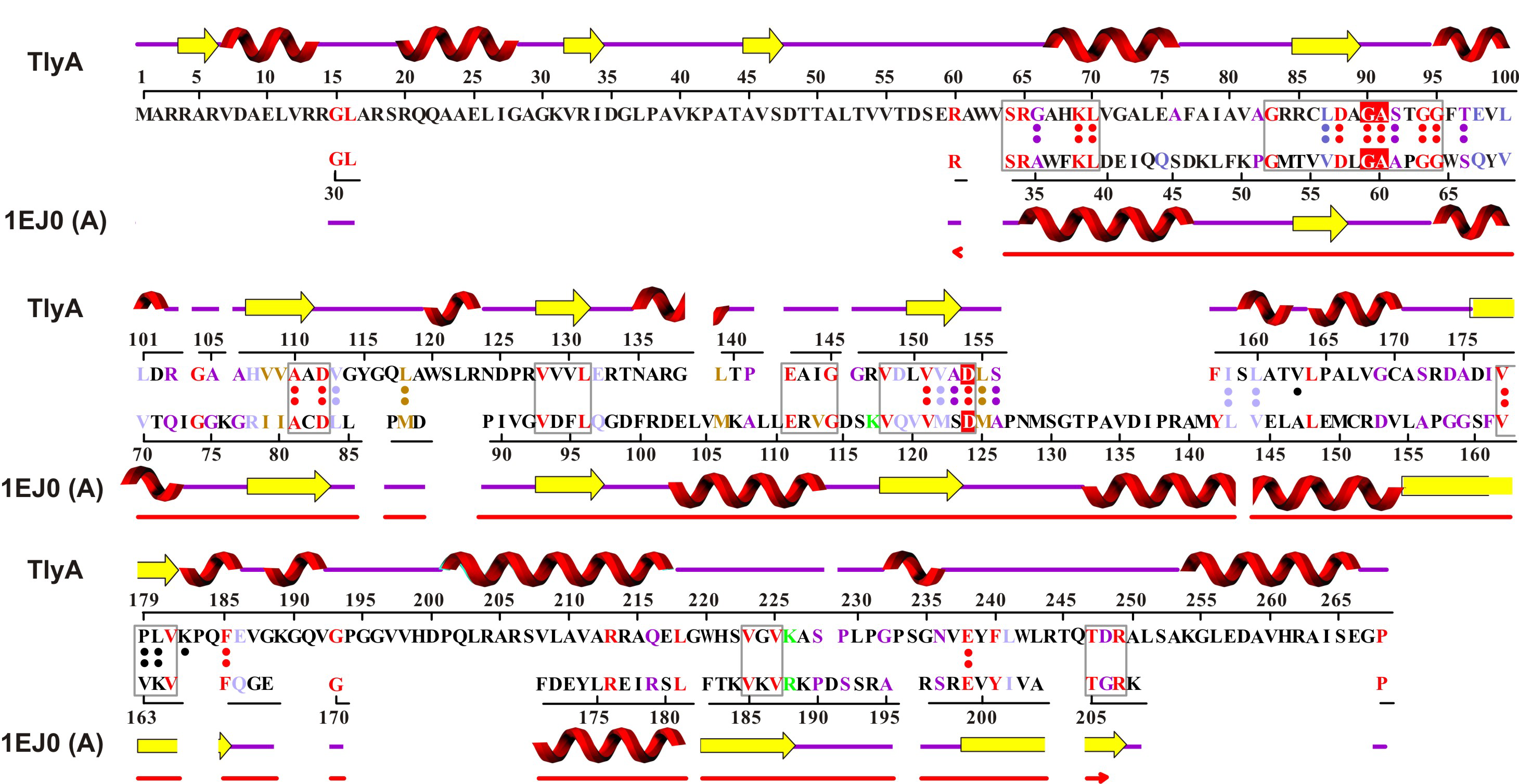

Supplement: Additional file 2 — Figure S1. Sequence alignment and secondary structure representation for TlyA and Ftsj-like RNA methyltransferase complexed with AdoMet, defined as the ligand-binding template [PDB:1EJ0] [31] showing the matched residues with the template. Alpha helices are represented by red spirals, beta strands by yellow arrows and random structures by straight lines. The red bar indicates the region with greater structural similarity (152 residues-long). Amino acid regions sharing the highest similarity are enclosed in boxes. [file 1472-6807-11-16-S2.TIFF]

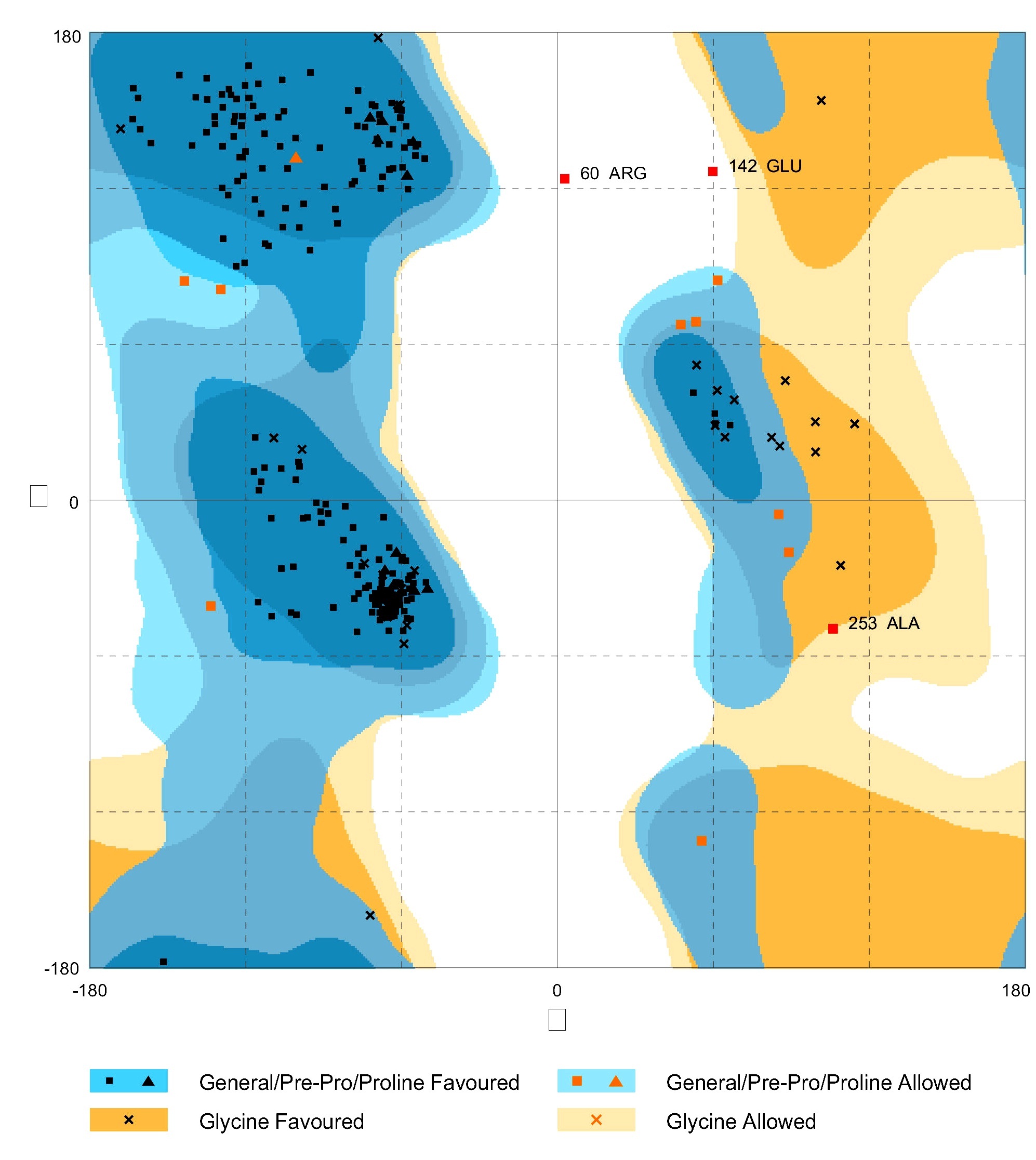

Supplement: Additional file 3 — Figure S2. Ramachandran plot of TlyA three-dimensional modeled structure. Dispersion zones are shown in blue and orange representing proline and glycine favored and allowed regions (Figure obtained by RAMPAGE server). [file 1472-6807-11-16-S3.JPEG]
